# Serum miR-96-5P and miR-339-5P Are Potential Biomarkers for Multiple System Atrophy and Parkinson's Disease

**DOI:** 10.3389/fnagi.2021.632891

**Published:** 2021-07-26

**Authors:** Annamaria Vallelunga, Tommaso Iannitti, Sabrina Capece, Gerardina Somma, Maria Claudia Russillo, Alexandra Foubert-Samier, Brice Laurens, Igor Sibon, Wassilios G. Meissner, Paolo Barone, Maria Teresa Pellecchia

**Affiliations:** ^1^Neuroscience Section, Department of Medicine and Surgery, Center for Neurodegenerative Diseases, University of Salerno, Salerno, Italy; ^2^Independent Researcher, Southampton, United Kingdom; ^3^Centre Hospitalier Universitarie, Service de Neurologie, CHU Bordeaux, Bordeaux, France; ^4^Universitè de Bordeaux, Institut des Maladies Neurodégénératives, UMR 5293, Bordeaux, France; ^5^CNRS, Institut des Maladies Neurodégénératives, UMR 5293, Bordeaux, France; ^6^Department of Medicine, University of Otago, Christchurch, New Zealand

**Keywords:** microRNAs, biomarkers, Parkinson's disease, multiple system atrophy, miR-96-5p, miR-339-5p

## Abstract

Parkinson's disease (PD) and Multiple System Atrophy (MSA) are progressive neurodegenerative diseases with overlap of symptoms in early stages of disease. No reliable biomarker exists and the diagnosis is mainly based on clinical features. Several studies suggest that miRNAs are involved in PD and MSA pathogenesis. Our goal was to study two serum circulating microRNAs (miR-96-5p and miR-339-5p) as novel biomarkers for the differential diagnosis between PD and MSA. Serum samples were obtained from 51 PD patients, 52 MSA patients and 56 healthy controls (HC). We measured levels of miRNAs using quantitative PCR and compared the levels of miR-96-5p and miR-339-5p among PD, MSA and HC groups using a one-way analysis of variance. Correlations between miRNA expression and clinical data were calculated using Pearson's rho test. We used the miRTarBase to detect miRNA targets and STRING to evaluate co-expression relationship among target genes. MiR-96-5p was significantly increased in MSA patients compared with HC (Fold change (fc): 3.6; *p* = 0.0001) while it was decreased in PD patients compared with HC (Fold change: 4; *p* = 0.0002). Higher miR-96-5P levels were directly related to longer disease duration in MSA patients. We observed a significant increase of miR-339-5p in MSA patients compared with PD patients (fc: 2.5; *p* = 0.00013). miR-339-5p was increased in MSA patients compared with HC (fc: 2.4; *p* = 0.002). We identified 32 target genes of miR-96-5p and miR-339-5p, some of which are involved in neurodegenerative diseases. The study of those miRNAs could be useful to identify non-invasive biomarkers for early differential diagnosis between PD and MSA.

## Introduction

Parkinson's disease (PD) and Multiple System Atrophy (MSA) are neurodegenerative disorders with overlap of symptoms, especially at early disease stages. However, they present different disease progression and response to dopaminergic treatment (Vallelunga et al., 2014a). The diagnosis is mostly based on clinical features while no serum biomarker is currently available (Laurens et al., 2015). Several reports support the hypothesis of overlapping molecular pathways between Alzheimer's disease (AD) and both MSA and PD. Many studies reported significant associations among detectable changes in cerebrospinal fluid (CSF), amyloid beta (Aβ) levels and clinical features of PD (Beyer et al., 2013; Kang et al., 2013; Alves et al., 2014). BACE1 is a promising target gene because is responsible for the cleavage of the amyloid precursor protein into amyloid-β fragments, a crucial step for the initiation of amyloid pathology (Peters et al., 2018). Lange et al. found an association of the rs638405 polymorphism of BACE1 with increased risk of PD in a Norwegian population (Lange et al., 2015). Furthermore, another research group identified a potential link between AD and MSA pathology, which involves the deregulation of microRNAs (miRNAs) and BACE1 (Kim et al., 2019). Several studies have identified minimally invasive biomarkers using body fluids such as serum, blood, plasma, urine and saliva. miRNAs are a class of endogenous RNAs, which regulate gene expression at post-transcriptional level (Ramaswamy et al., 2018). Many studies suggest that miRNAs can cross the blood brain barrier and enter body fluids such as blood (Kumar and Reddy, 2016). Circulating miRNAs (cmiRNAs) are highly stable, easily quantifiable and accessible by minimally-invasive procedures. Change in the expression of many cmiRNAs is associated with pathophysiological processes relevant to PD pathology. Several reports suggest the utility of cmiRNAs as potential biomarkers for PD and other parkinsonian disorders (Ubhi et al., 2014; Schafferer et al., 2016; Huang et al., 2019; Vallelunga et al., 2019). Many miRNAs regulate the expression of BACE 1 but miR-339-5p directly interacts with the 3′-UTR of BACE-1 and deregulates its expression and biological activity (Rahmani et al., 2019). Long et al. reported that miR-339-5p regulates BACE-1 in human brain cells and it is dysregulated in a subset of AD patients (Long et al., 2014). In a previous study, we found a decrease of serum miR-339-5p in 25 MSA and 25 PD patients compared with 25 healthy controls (HC) (Vallelunga et al., 2014b). MiR-96-5p has also shown promise as a potential biomarker. Ubhi et al. reported an increase of miR-96 in human brain MSA samples and in a mouse model of MSA (Ubhi et al., 2010). Furthermore, they observed that target genes of miR-96-5p were decreased in MSA patients and a mouse model of MSA (Virachit et al., 2019). In addition, Schafferer et al. found an increase of miR-96 in the later stages of MSA in both the mouse model and human MSA samples (Schafferer et al., 2016). Recently, Vidal-Martinez et al. reported that FTY720-Mitoxy, a FTY720-derivative known to increase expression of oligodendrocyte brain-derived neurotrophic factor (BDNF), glial cell line-derived neurotrophic factor (GDNF), and nerve growth factor (NGF), reduces brain miR-96-5p (Vidal-Martinez et al., 2020). In the present study, our primary aim was to investigate serum levels of miR-339-5p and miR-96-5p from MSA and PD patients compared to HC. Our secondary aim was to assess whether miR-339-5p and miR-96-5p expression could differentiate PD from MSA patients.

## Methods

### Study Design and miRNAs Quantification

In a previous study (Vallelunga et al., 2014b), we detected differences between selected miRNAs between MSA and PD patients using a sample of 25 patients in each group. Based on the findings from our previous investigation, in the present study, we recruited 51 PD and 52 MSA patients diagnosed according to the criteria currently used in the clinic (Gilman et al., 2008; Postuma et al., 2015). We included 56 HC with no history of neurological or psychiatric diseases. Serum samples from PD and MSA patients were collected at the Center for Neurodegenerative Diseases at the University of Salerno (Salerno, Italy), and the Department of Neurology at the Expert Center for PD and French Reference Center for MSA at the University Hospital of Bordeaux (Bordeaux, France). Serum samples from HC were also collected at the institutions mentioned above. The study protocol was approved by the institutional review board at the University of Salerno (Salerno, Italy) and the CPP Sud-Ouest et Outre-Mer III (Bordeaux, France). Informed consent was obtained from all participants. Demographic and clinical features are reported in the Supplementary Table 1. Blood samples were obtained by vein puncture using dry vacutainer tubes (BD Biosciences, Italy). Each sample was processed for serum isolation within 2 h after withdrawal according to the protocol described below. Whole blood was left to stand for about 30 min at 20°C before being centrifuged at 3,000 rpm for 15 min at 4°C. The serum was divided into aliquots and stored at −80°C until analysis. Before analyzing the serum samples, we assessed hemolysis according to Shah et al. (2016), which determined the ratio of miR-451a (ID: 001105; Thermo Fisher Scientific, Italy) to miR-23a-3p (ID: 0003999; Thermo Fisher Scientific, Italy) to determine the samples with low (miR ratio < 5), moderate (5 < miR ratio > 7) and severe (miR > 7) grade of hemolysis. We then proceeded to exclude all samples with moderate or severe hemolysis. Serum miR-339-5p and miR-96-5p were quantified using LNA^™^ enhanced microRNA assay (Exiqon) according to our previous published protocol (Vallelunga et al., 2019). Each miRNA was quantified in duplicate and mean Ct values were used for fold change calculations. The identification numbers of miRNA assays (probes) used for RT-qPCR are as follows: has-miR-93-5p (204715), has-miR-339-5p (206007) and has-miR-96-5p (204417).

### Data Analysis and Statistics

We normalized the data using miR-93-5p as reference miRNA (Vallelunga et al., 2019). To establish if miR-93-5p was an appropriate normalizer, we analyzed miR-93-5p in our cohorts of patients and HCs. This data was normally distributed as assessed by D'Agostino and Pearson test and was analyzed using a one-way analysis of variance (ANOVA), which showed no difference among PD, MSA and HC subjects divided by sex (Supplementary Figure 1). We considered 37 as a cut off value for Cts and we excluded samples with Ct values higher than 37 from the analysis. We calculated the fold changes (fc) using the 2^−ΔCT^ method for miR-339-5p and miR-96-5p. These data were not normally distributed as assessed by D'Agostino and Pearson test and were analyzed using a Mann-Whitney test for comparison of miRNA expression levels among PD, MSA and HC groups. Correlations between miRNA expression and disease duration were assessed using the Spearman's correlation test. To assess the diagnostic accuracy of miRNAs to discriminate among PD and MSA patients, we performed a receiver operating characteristic curve (ROC). All statistical analyses were performed using GraphPad Prism (GraphPad Software Inc., San Diego, CA, USA) except for the ROC analyses, which were performed using MedCalc Software Version 19.6.4 (Ostend, Belgium). A *p* < 0.05 was considered significant.

### Target Prediction

Target prediction of miR-96-5p and miR-339-5p was obtained querying the microRNA-target interactions using the miRTarBase, chosen due to its widespread use and completeness. We considered only strong mRNA-miRNA interactions experimentally confirmed by qRT-PCR, luciferase assays and Western Blots. Then, we used the search tool for retrieval of interacting genes (STRING) to evaluate co-expression relationships among target genes. We considered only the target genes with co-expression coefficients >0.7.

## Results

### Serum miR-96-5p

**Figure 1:**
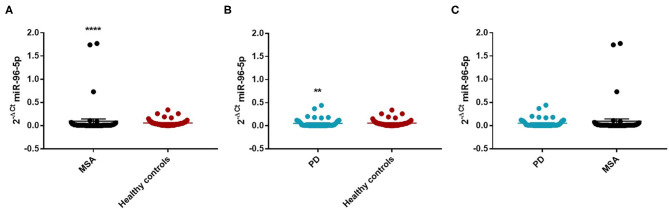
Comparison of 2^−ΔCT^ values for serum miR-96-5p between MSA and HC **(A)**, PD and HC **(B)**, and PD and MSA **(C)** subjects. ^**^p < 0.01 and ^****^p < 0.0001.

We observed that miR-96-5p was significantly increased in MSA (Mean ± SEM:0.096 ± 0.048) patients compared with HC (Mean ± SEM:0.057 ± 0.009) (fc: 1.61; *p* < 0.0001) (Figure 1A).

**Figure 2:**
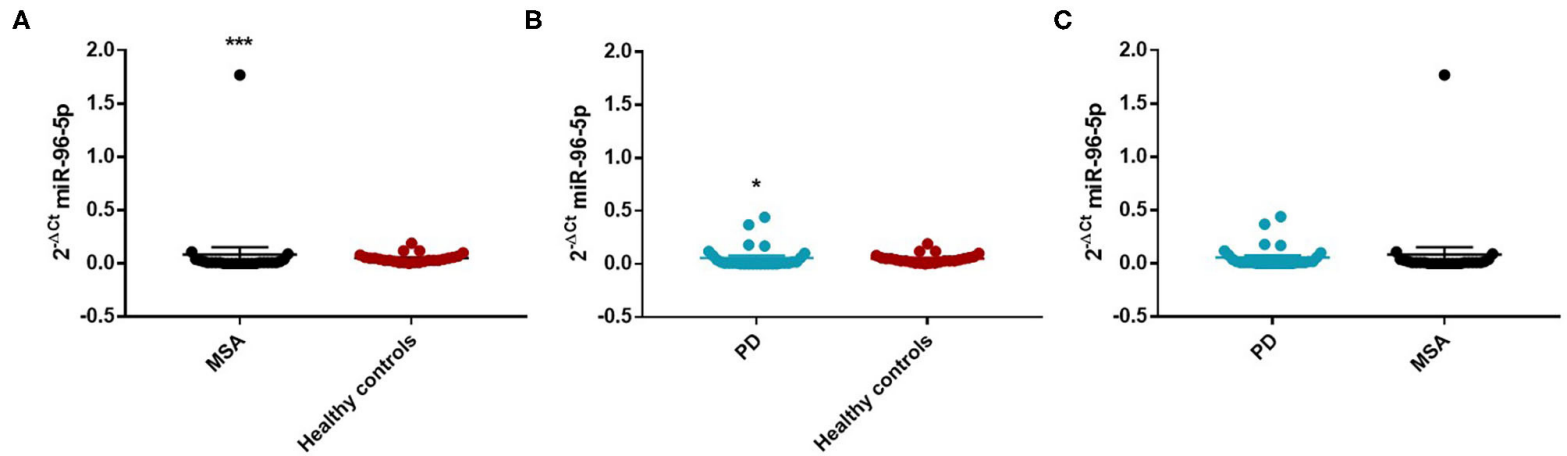
Comparison of 2^−ΔCT^ values for serum miR-96-5p between male MSA and HC **(A)**, PD and HC **(B)**, and PD and MSA **(C)** subjects. ^*^p < 0.05 and ^***^p < 0.001.

We performed a ROC analysis to establish the diagnostic accuracy of miR-96-5p and we observed that this miRNA discriminated well between MSA and HC [area under the curve (AUC) = 0.736; *p* < 0.001] with a sensitivity of 77.4% and a specificity of 67.3% (Supplementary Figure 2). Similarly, miR-96-5p expression was decreased in PD patients (Mean ± SEM:0.051 ± 0.012) compared with HC (Mean ± SEM:0.057 ± 0.009) (fc:0.89; *p* < 0.01) (Figure 1B). The AUC for miR-96-5p, when comparing PD and HC, was 0.645 (Supplementary Figure 3). We did not observe significant differences in miR-96-5p levels when comparing PD and MSA patients (fc:1.1; *p* = 0.74) (Figure 1C). When analyzing male and female miR-96-5p data separately, we found an increase of miR-96-5p in female MSA patients (fc:1.62; *p* < 0.05) (Supplementary Figure 4A) and male counterparts (Mean ± SEM:0.085 ± 0.067) (fc:1.65; *p* < 0.001) (Figure 2A) compared with HC female and male subjects (Mean ± SEM:0.05 ± 0.008), respectively. Furthermore, we observed a significant miR-96-5p increase only when comparing male PD subjects (Mean ± SEM:0.058 ± 0.02) to sex-matched HC subjects (Mean ± SEM:0.05 ± 0.08) (fc:1.17; *p* < 0.05) (Figure 2B). No significant differences were observed when comparing MSA and PD male patients (Figure 2C). The results of the ROC analyses for this miRNA are reported in the Supplementary Materials and indicate that miR-96-5p is moderately accurate in the context of our comparisons. Nevertheless, when comparing MSA and HC male patients, we observed a high specificity (92%) and moderate sensitivity (63%) (Supplementary Figures 5–7). No significant differences were observed for miR-96-5p when comparing MSA-P with MSA-C (Supplementary Figure 8B). No correlation was found between miR-96-5p levels and disease duration in both PD and MSA patients of both sexes (Supplementary Figures 9, 10). Using miRTarBase, we identified 203 target genes of miR-96-5p but only 27 of them had experimentally confirmed strong mRNA-miRNA interactions. Many predicted target genes are involved in neurodegeneration. By using STRING, we found several interactions among target proteins (Supplementary Figure 11). We observed that 12 target genes are involved in the regulation of apoptotic processes [False Discovery Rate (FDR) = 2.66e-05]. In addition, 10 target genes are implicated in neurogenesis (FDR = 0.00069) and seven genes are involved in the regulation of neurogenesis (FDR = 0.0012). Other target genes are implicated in the regulation of dopamine receptor signaling. These results suggest that these genes work together in different pathways involved in neurodegeneration such as neuronal survival and dopaminergic signaling (Supplementary Table 2).

### Serum miR-339-5p

**Figure 3:**
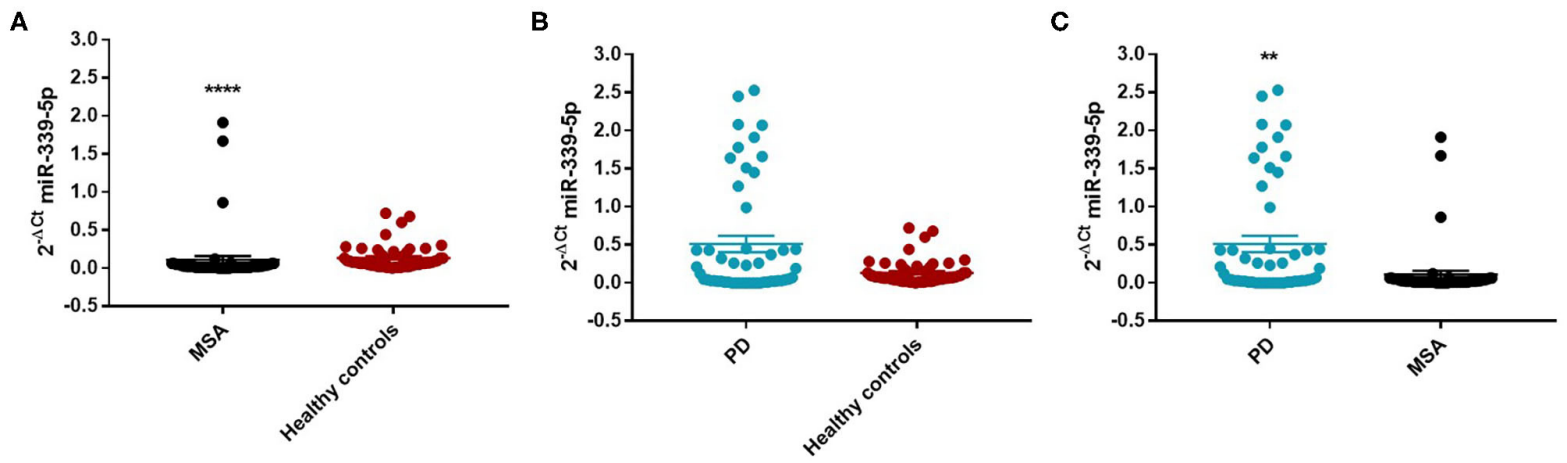
Comparison of 2^−ΔCT^ values for serum miR-339-5p between MSA and HC **(A)**, PD and HC **(B)**, and PD and MSA **(C)** subjects. ^****^p < 0.0001 and ^**^p < 0.01.

We found an decrease of miR-339-5p in MSA patients (Mean ± SEM:0.107 ± 0.049) compared with HC (Mean ± SEM:0.132 ± 0.021) (fc: 0.80; *p* < 0.0001) (Figure 3A).

**Figure 4:**
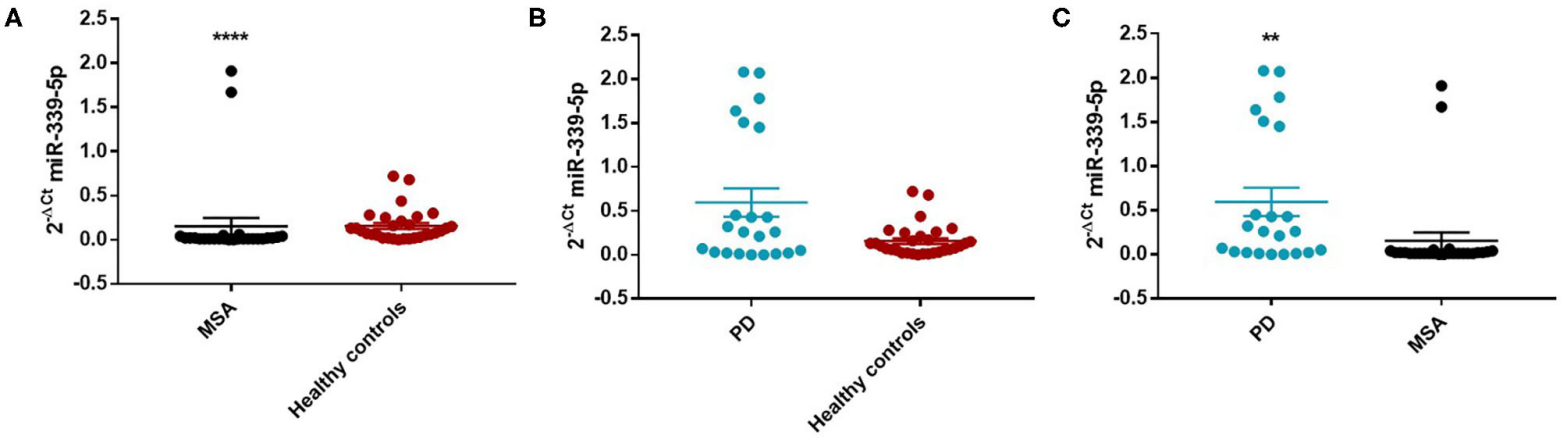
Comparison of 2^−ΔCT^ values for serum miR-339-5p between female MSA and HC **(A)**, PD and HC **(B)**, and PD and MSA **(C)** subjects. ^****^p < 0.0001 and ^**^p < 0.01.

Similarly, we observed a decrease of miR-339-5p in MSA female (Mean ± SEM:0.154 ± 0.094) (fc:0.98; *p* < 0.0001) (Figure 4A) and male (fc:0.6; *p* < 0.001) patients (Supplementary Figure 12A) compared with sex-matched HC (Mean ± SEM:0.156 ± 0.033). No differences were observed in PD patients compared with HC in regards to miR-339-5p for both male and female patients (Figures 3B and 4B).

We observed a significant increase of miR-339-5p in PD patients (Mean ± SEM:0.509 ± 0.105) compared with MSA patients (Mean ± SEM:0.107 ± 0.049) (fc:4.78; *p* < 0.01) (Figure 3C). To determine the diagnostic accuracy of miR-339-5p to discriminate between PD and MSA, a ROC analysis was performed. The specificity of miR339-5p to discriminate between MSA and PD was 90.6% but the sensitivity was 51% (Supplementary Figure 13). When analyzing male and female miR data separately, we found a significant increase of miR-339-5p only in PD female patients (Mean ± SEM:0.595 ± 0.160) compared with MSA female subjects (Mean ± SEM:0.154 ± 0.094) (fc:3.84; *p* < 0.01) (Figure 4C). The specificity of miR339-5p to discriminate between PD and MSA female subjects was 88%, and the sensitivity was 68.8% (Supplementary Figure 14). A significant correlation was observed between disease duration in female PD patients and mir-339-5p (*r* = −0.783; *p* < 0.0001) (Supplementary Figure 9A) and disease duration in male PD subjects and mir-339-5p (*r* = −0.773; *p* < 0.0001) (Supplementary Figure 9B). No significant differences were observed for miR-339-5p when comparing MSA-P with MSA-C (Supplementary Figure 8A). No correlation was found between miR-339-5p and disease duration in both female (Supplementary Figure 10A) and male MSA patients (Supplementary Figure 10B). By using miRTarBase database, we identified 237 target genes but only 5 of them showed experimentally confirmed strong mRNA-miRNA interactions (BACE1, NOVA1, MDM2, BCL6 and PTP4A1). Moreover, we observed that other target genes with weak predicted functionality could be involved in the pathogenesis of MSA (i.e., AMBRA1, CTSD, ELOVL7 and HNRNPA1). No relationship among target genes was identified.

## Discussion

In our study, we found a significant increase of serum miR-96-5p in MSA patients compared to HC while in PD patients we observed a decrease compared to HC. Our results are consistent with the increased miR-96-5p expression in the transgenic myelin basic protein (MBP) mouse model of MSA and post-mortem brain tissue from MSA patients (Ubhi et al., 2014; Schafferer et al., 2016) while miR-96-5p has not previously been studied in PD. Ubhi et al. found a decrease of miR-96 target genes in human MSA brain and transgenic MBP mice pointing to a possible involvement of this miRNA in MSA pathogenesis (Ubhi et al., 2014). Recently, Schafferer et al. also observed that miR-96-5p was increased in brain tissue from MSA patients but not in the early pre-symptomatic stage in the transgenic proteolipid protein mouse model of MSA (Schafferer et al., 2016). This difference may be related to changes in the miRNA profile as disease progresses, which may reflect different events in the early and late stages of MSA. Recently, Vidal-Martinez et al. observed that levels of miR-96-5p were significantly reduced in FTY720-Mitoxy treated aSyn Tg mice that display MSA-like impairments. Furthermore, they confirmed that FTY720-Mitoxy increases neuronal BDNF and oligodendroglial GDNF, BDNF and NGF expression. For the above reasons, FTY720-Mitoxy could be a potential therapeutic agent for MSA that can improve motor function while decreasing synucleinopathy. The beneficial effects of this therapeutic agent were associated with reduced miR-96-5p expression and a corresponding increase in the neurotrophic factor GDNF (Vidal-Martinez et al., 2020). Using miRTarBase, we predicted several target genes of miR-96-5p and hypothesize that an increase of this miRNA can lead to decrease of predicted target genes. We identified 203 target genes of miR-96-5p using miRTarBase and used STRING to determine protein interactions among those genes involved in key biological processes such as regulation of apoptosis, dopaminergic signaling and neurogenesis. Many predicted target genes are involved in neuronal survival and synaptic plasticity and have a neuroprotective function such as BDNF, GDNF and tissue inhibitor of metalloproteinases-1 (TIMP-1). Other target genes may be specifically involved in the pathogenesis of MSA such as SLC1A1 and SLC6A6 (Ubhi et al., 2010). Recently, Huang et al. observed a significant reduction of BDNF expression in peripheral blood lymphocytes of PD patients. In addition, they found that BDNF levels were associated with disease duration, Unified Parkinson's Disease Rating Scale score, H-Y staging and treatment with L-DOPA (Lorenzl et al., 2003). Rahmani et al. conducted a meta-analysis to study if serum/CSF levels of BDNF change in patients with PD. All studies considered in this meta-analysis reported a significant reduction of BDNF in PD patients' CSF and serum compared to HC (Shi et al., 2015). Recently, Virachit et al. observed a significant reduction in hippocampal levels of GDNF in PD patients compared to HC (Virachit et al., 2019). Ubhi et al. quantified GDNF levels in several brain regions of MSA patients. They found a 50% reduction in frontal cortex white matter and cerebellum of MSA patients compared with HC (Ubhi et al., 2010). Conflicting data were reported for TIMP-1 levels in PD patients. For instance, Lorenz et al. found an increase of TIMP-1 levels in CSF from PD patients (Lorenzl et al., 2003). On the contrary, Shi et al. showed a reduced TIMP-1 concentration in CSF of PD patients (Shi et al., 2015). Selective loss of SLC1A1 has been implicated in the cerebellar form of MSA (Dirson et al., 2002). Ubhi et al. observed a significant decrease in SLC1A1 mRNA in brain tissues from MSA patients and in transgenic MBP mice (Ubhi et al., 2014). SLC6A6, a taurine transporter with a neuroprotective role, was significantly reduced in MSA patients' frontal cortex (Ubhi et al., 2014). In the present study we also observed a significant increase of miR-339-5p in MSA compared to PD patients suggesting that it could represent a potential biomarker to differentiate MSA from PD. In regards to diagnostic accuracy of miR-339-5p to discriminate between PD and MSA patients, it is worth noting that the ROC analysis showed a high specificity (90.6%) to discriminate between MSA and PD although the sensitivity was 51%. This high specificity was also observed when analyzing female miR339-5p data (88%). In this study, we confirm that miR-339-5p levels are lower in MSA vs. HC in line with our previous publication (Vallelunga et al., 2014b). Additionally, no deregulation was observed in either of the articles when comparing PD to controls. In the current study, we observed an increase in miR-339-5p in PD vs. MSA patients. This may be due to the increased number of patients involved in the present study. In addition, the cohorts of patients we enrolled in our two studies present different clinical characteristics, which may also explain differences between the findings from our two studies. We identified 237 targets of miR-339-5 but only 5 genes showed a strong mRNA-miRNA interaction (BACE1, NOVA1, MDM2, BCL6 and PTP4A1). These are implicated in different neurodegenerative diseases. Intriguingly, we found that some predicted genes with weak functionality were involved in MSA pathogenesis including AMBRA1 and Cathepsin D (CTDS). Miki et al. observed an association between AMBRA1 and the native α-synuclein in MSA patients' brains. In addition, they found a decrease in levels of native α-synuclein binding to AMBRA1 in the cerebral cortex or cerebellar white matter of MSA patients compared with HC (Miki et al., 2018). Recently, Kiely et al. (2019) observed that CTDS activity was significantly increased in multiple brain regions of MSA patients. Further studies in larger cohorts of patients are necessary to confirm our findings and to take a step toward a minimally-invasive biomarker-based diagnosis of MSA and PD.

## Data Availability Statement

The raw data supporting the conclusions of this article can be made available by the authors upon request.

## Ethics Statement

The studies involving human participants were reviewed and approved by the institutional review board at the University of Salerno and the CPP Sud-Ouest et Outre-Mer III in Bordeaux. The patients/participants provided their written informed consent to participate in this study.

## Author Contributions

AV: conceptualization, methodology, writing of the article, investigation, and formal analysis. TI: conceptualization, investigation, formal analysis, and writing of the article. SC and GS: investigation. MR, AF-S, BL, and IS: resources. WM: writing of the article and review and editing of the article. PB: supervision. MP: writing of the article, review and editing of the article, and supervision. All authors approved the version submitted for publication.

## Conflict of Interest

The authors declare that the research was conducted in the absence of any commercial or financial relationships that could be construed as a potential conflict of interest.

## Publisher's Note

All claims expressed in this article are solely those of the authors and do not necessarily represent those of their affiliated organizations, or those of the publisher, the editors and the reviewers. Any product that may be evaluated in this article, or claim that may be made by its manufacturer, is not guaranteed or endorsed by the publisher.
