# Supplementary material for: Serum miR-96-5P and miR-339-5P Are Potential Biomarkers for Multiple System Atrophy and Parkinson's Disease
Source: Front Aging Neurosci. 2021 Jul 26;13:632891. doi: 10.3389/fnagi.2021.632891 (PMC8350521; doi:10.3389/fnagi.2021.632891)
Supplement: Supplementary file 1 [file Presentation_1.pptx]

## Slide 1
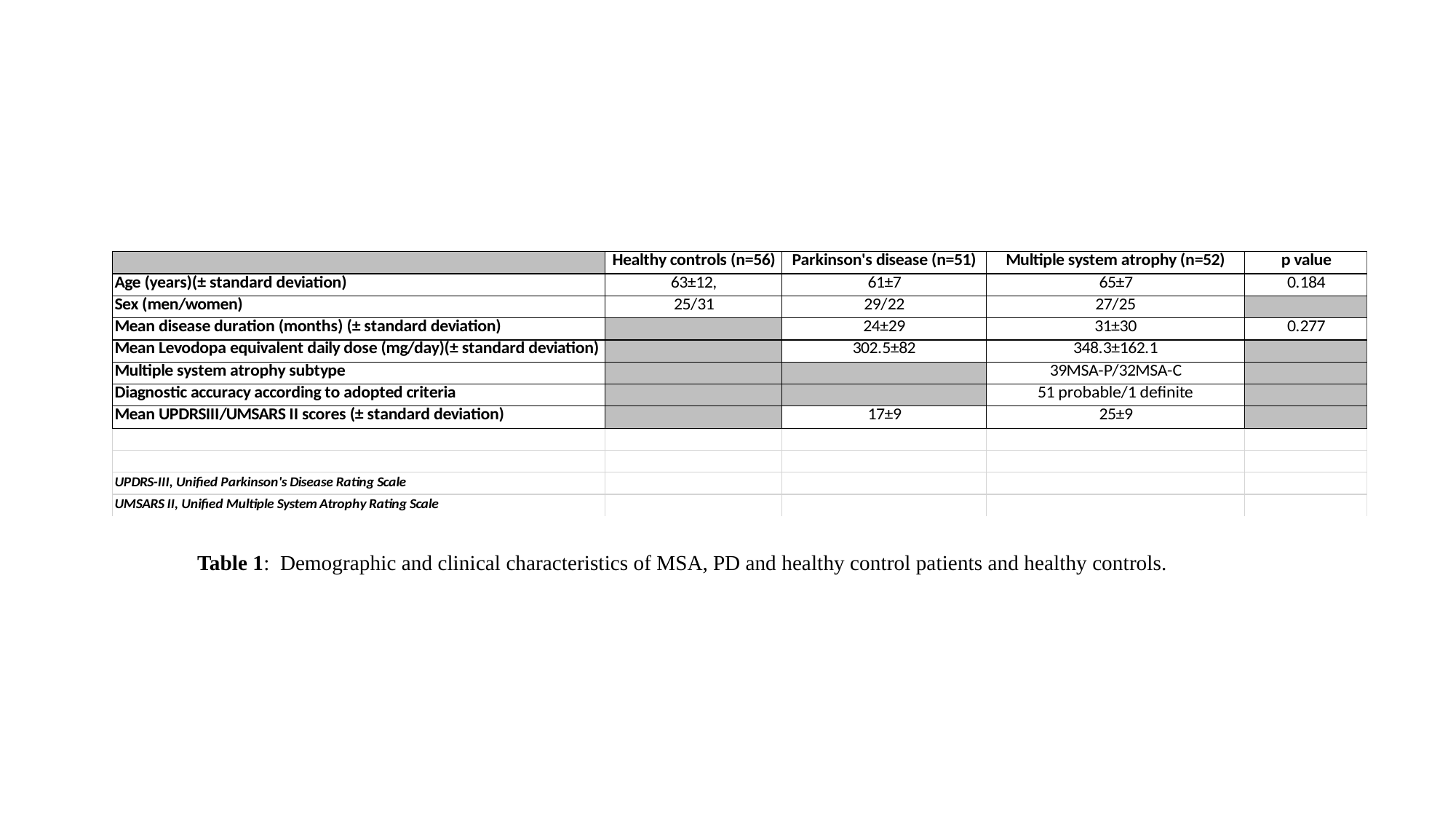

Table 1: Demographic and clinical characteristics of MSA, PD and healthy control patients and healthy controls.

## Slide 2
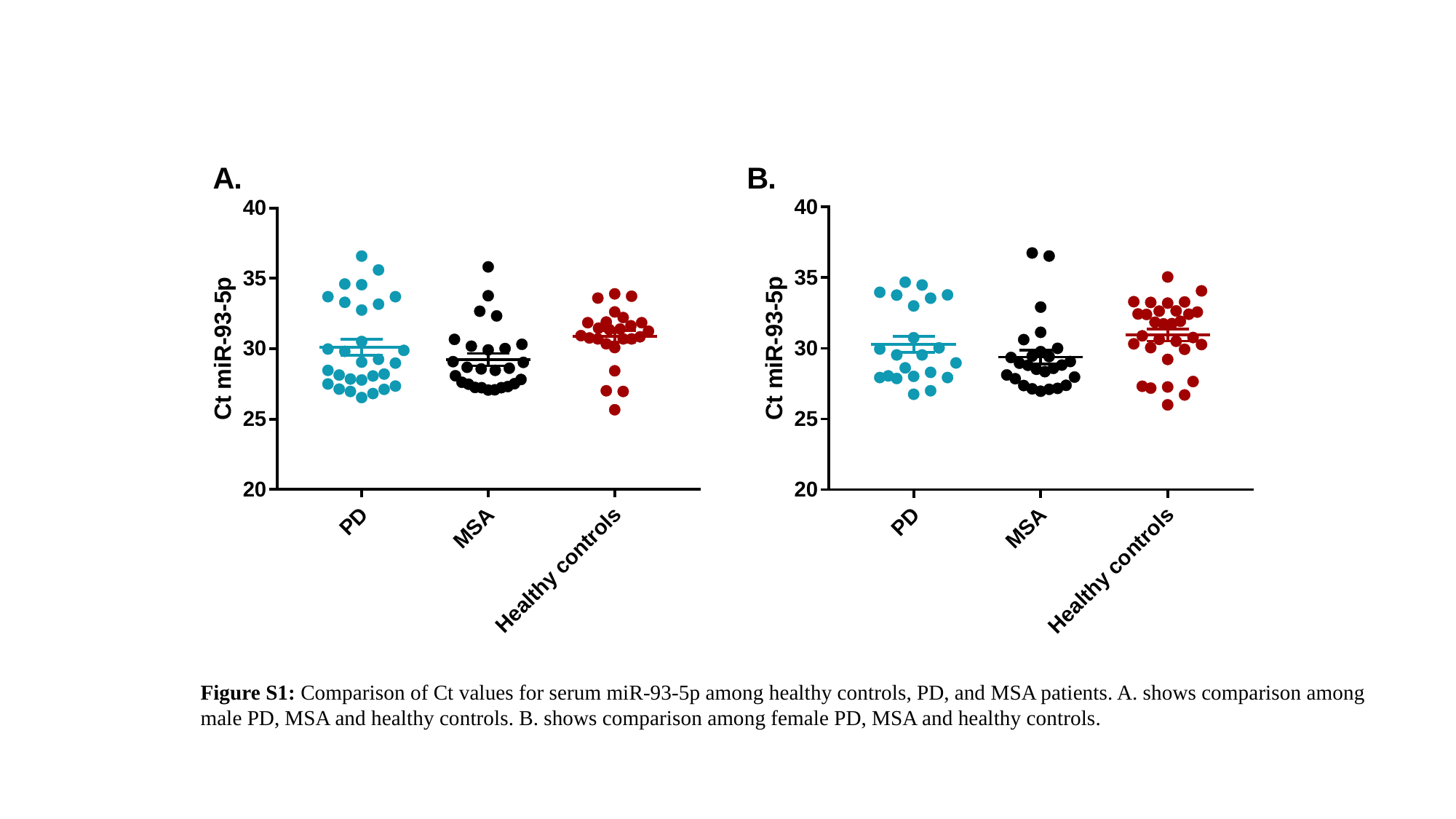

Figure S1: Comparison of Ct values for serum miR-93-5p among healthy controls, PD, and MSA patients. A. shows comparison among male PD, MSA and healthy controls. B. shows comparison among female PD, MSA and healthy controls.

## Slide 3
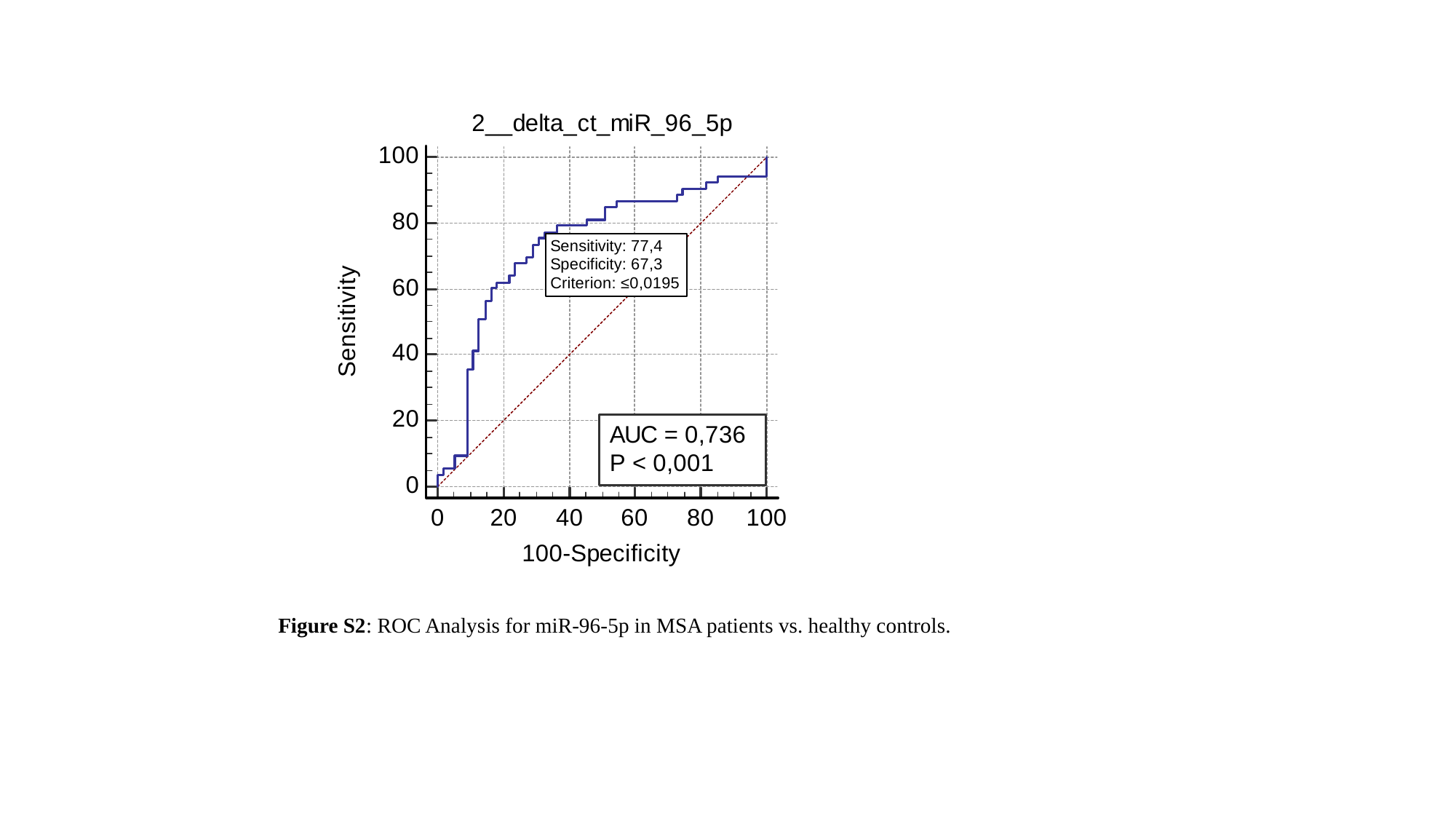

Figure S2: ROC Analysis for miR-96-5p in MSA patients vs. healthy controls.

## Slide 4
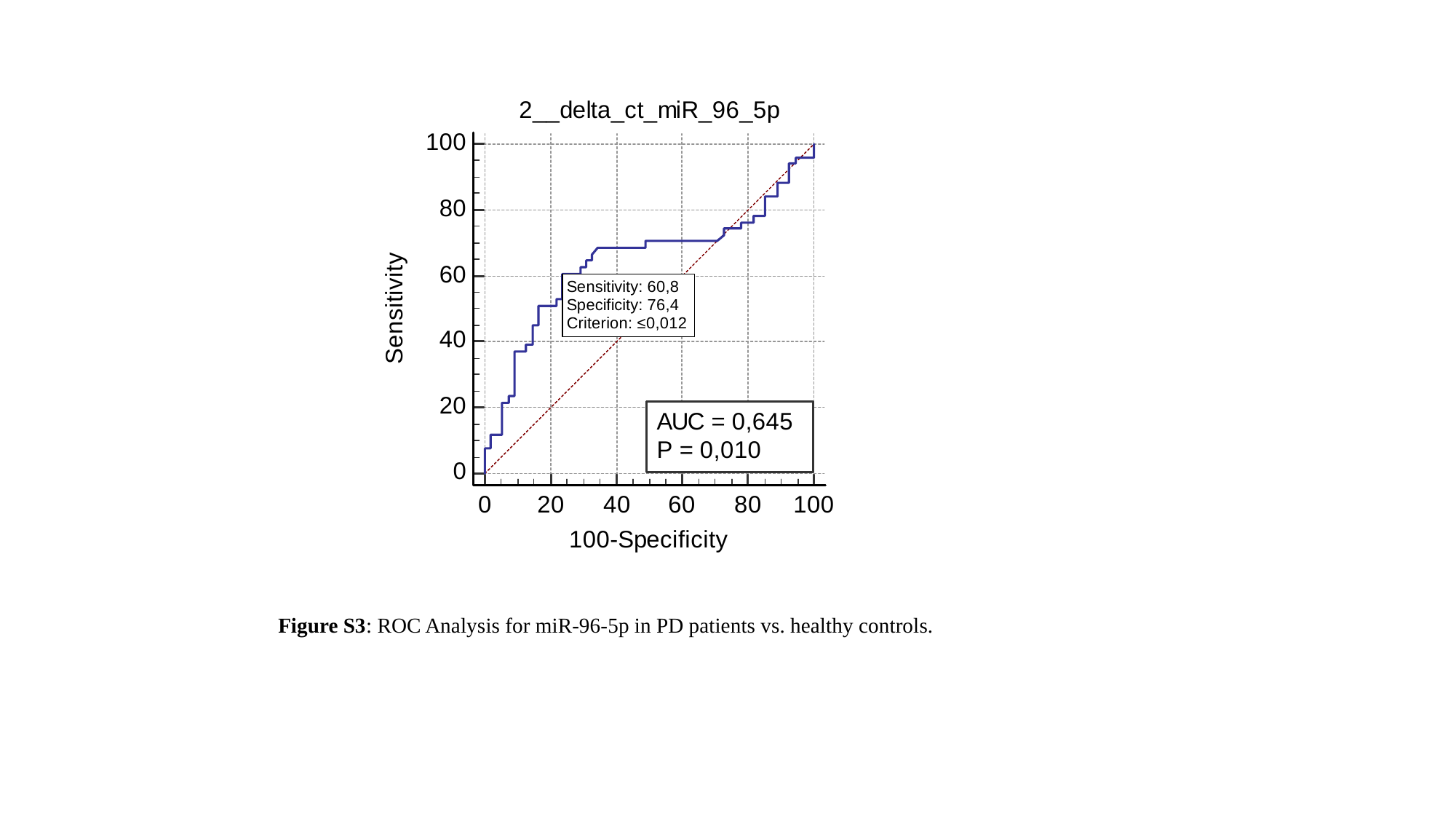

Figure S3: ROC Analysis for miR-96-5p in PD patients vs. healthy controls.

## Slide 5
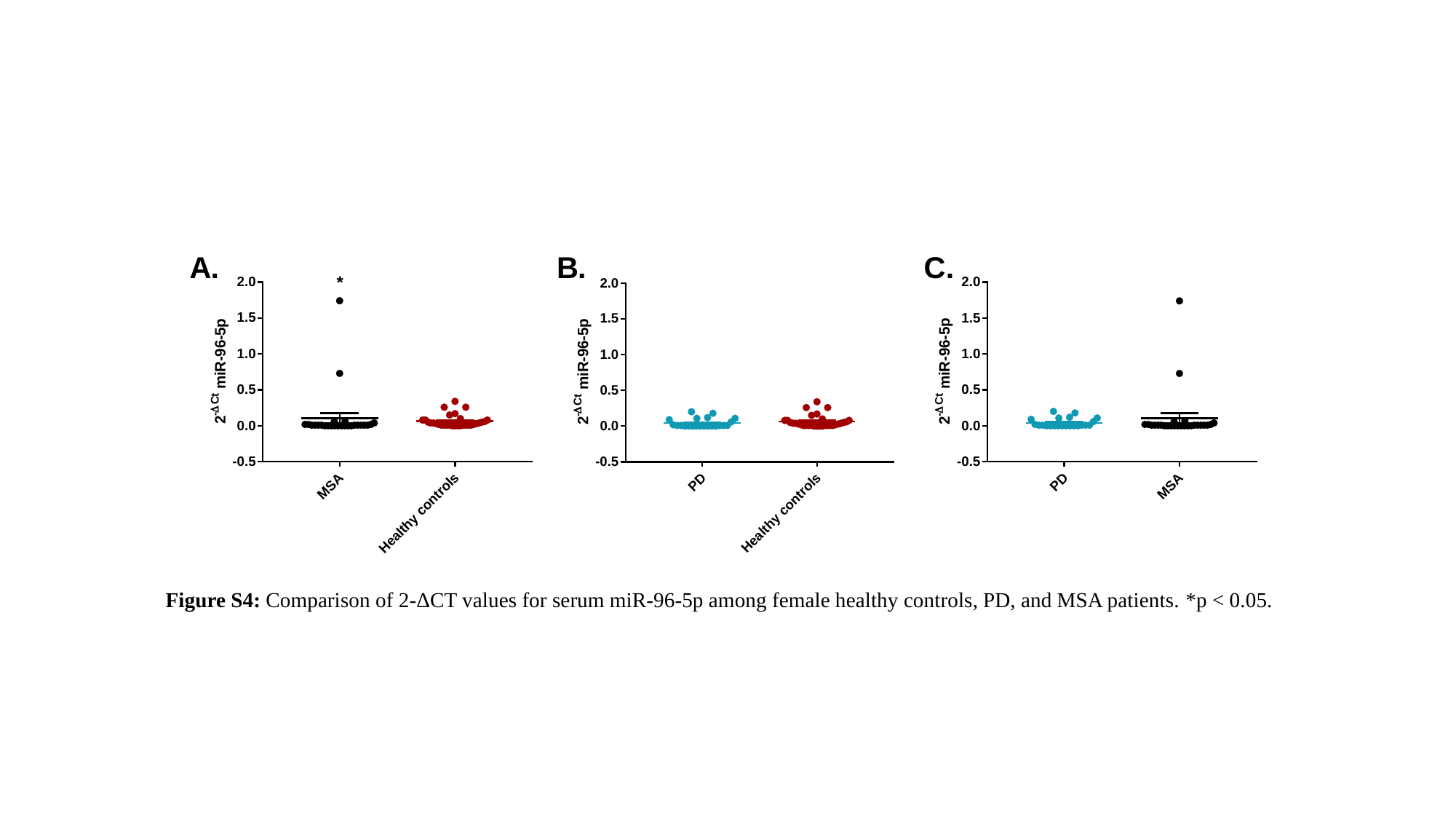

Figure S4: Comparison of 2-ΔCT values for serum miR-96-5p among female healthy controls, PD, and MSA patients. *p < 0.05.

## Slide 6
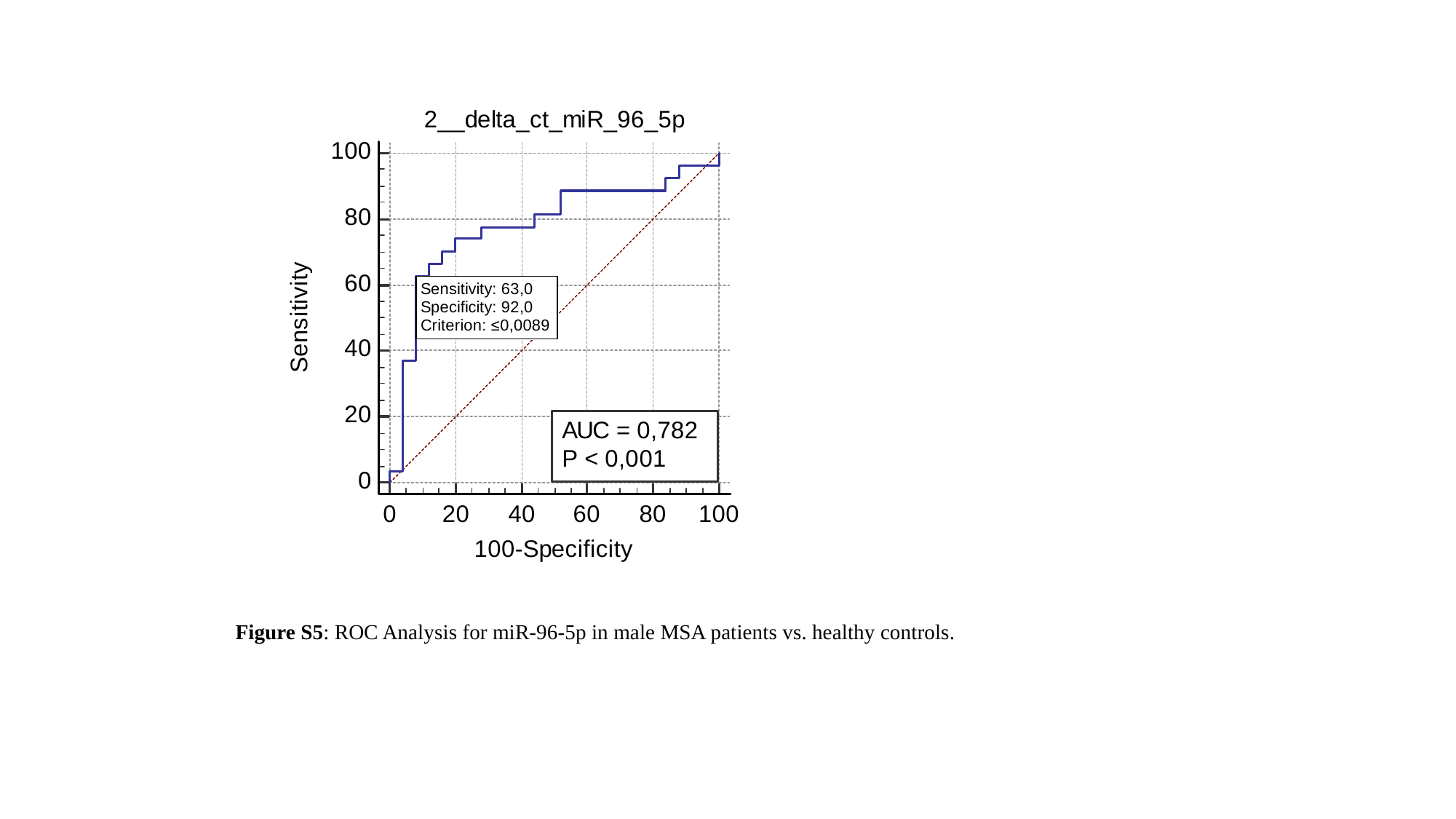

Figure S5: ROC Analysis for miR-96-5p in male MSA patients vs. healthy controls.

## Slide 7
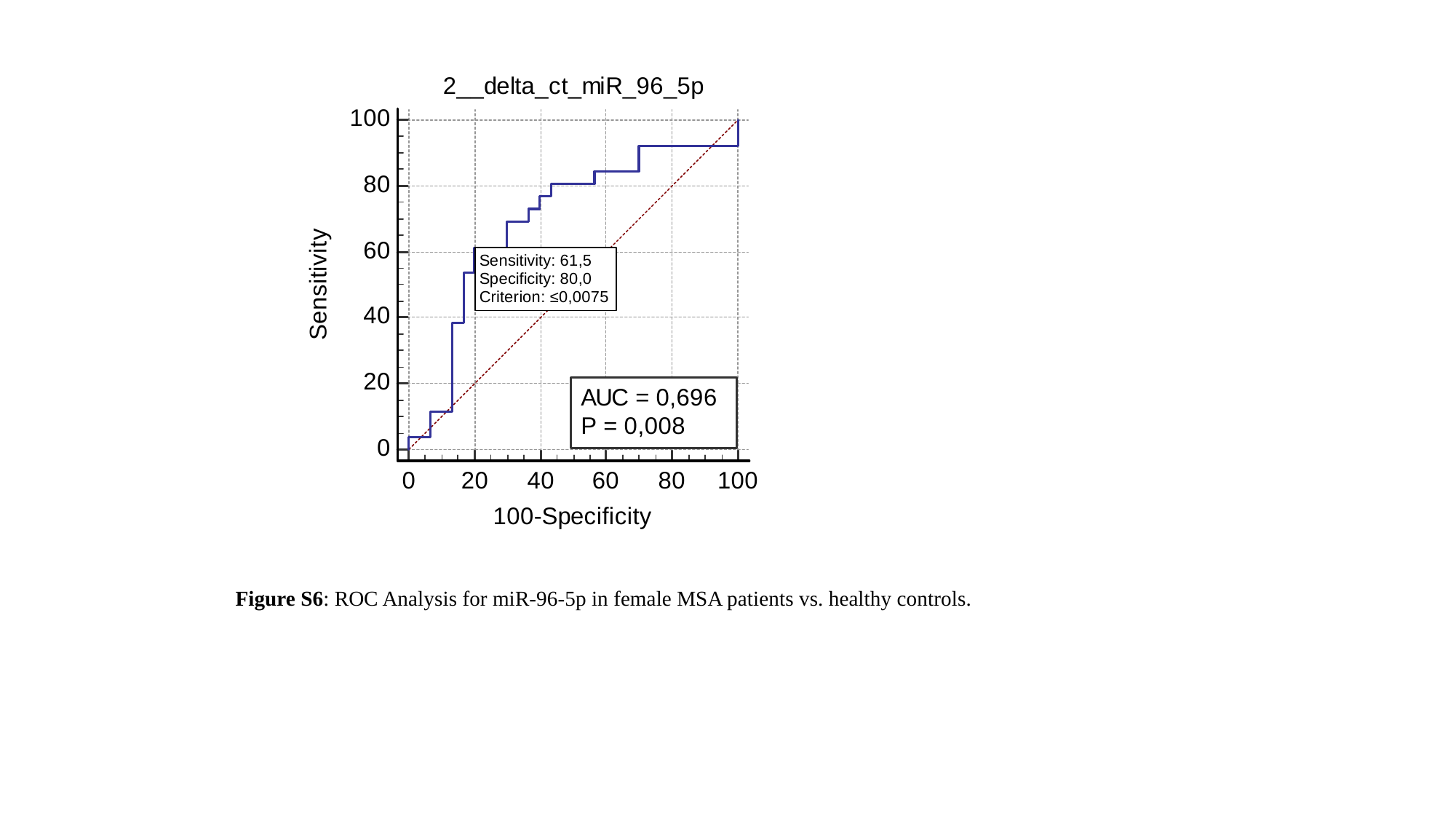

Figure S6: ROC Analysis for miR-96-5p in female MSA patients vs. healthy controls.

## Slide 8
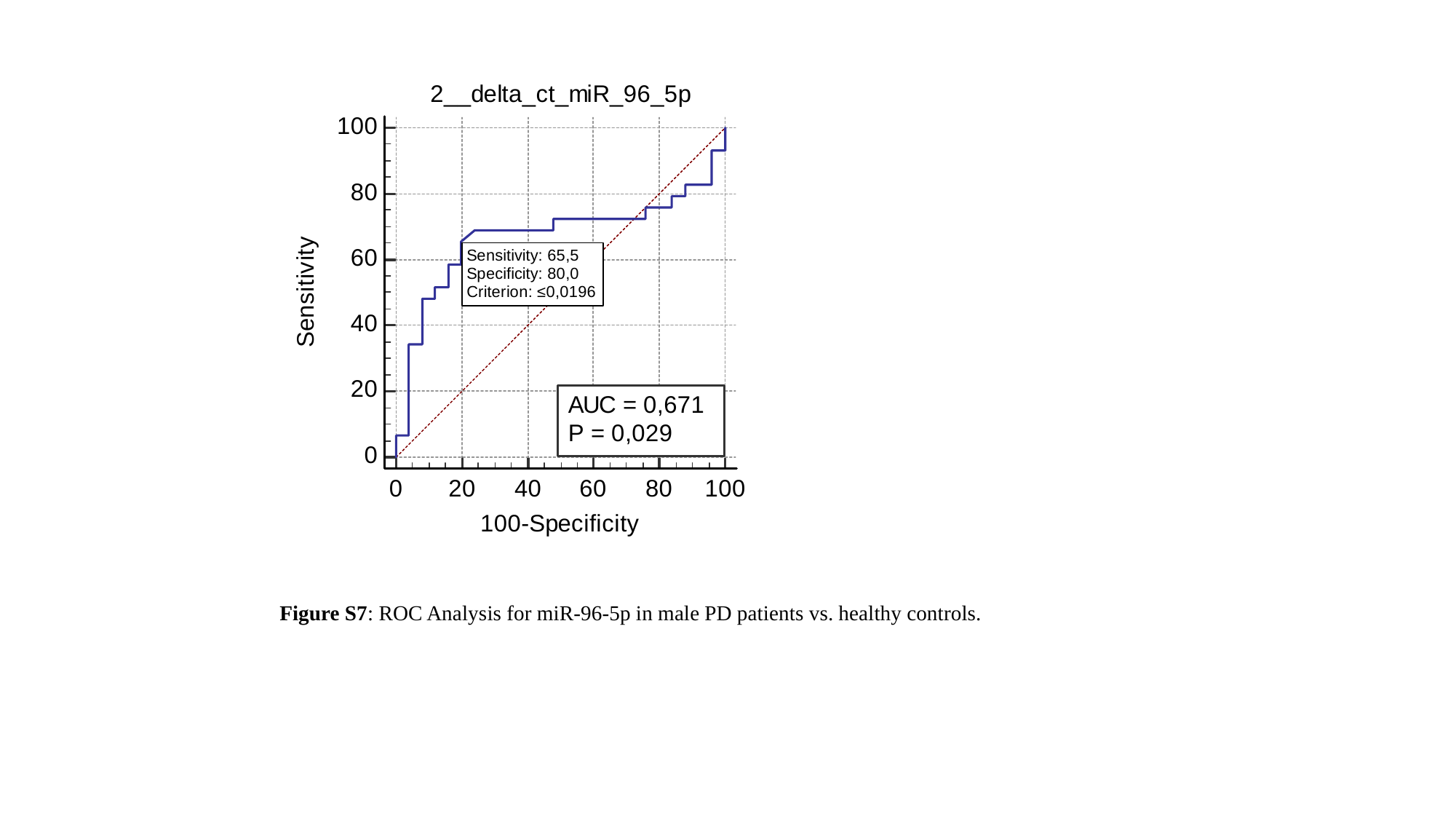

Figure S7: ROC Analysis for miR-96-5p in male PD patients vs. healthy controls.

## Slide 9
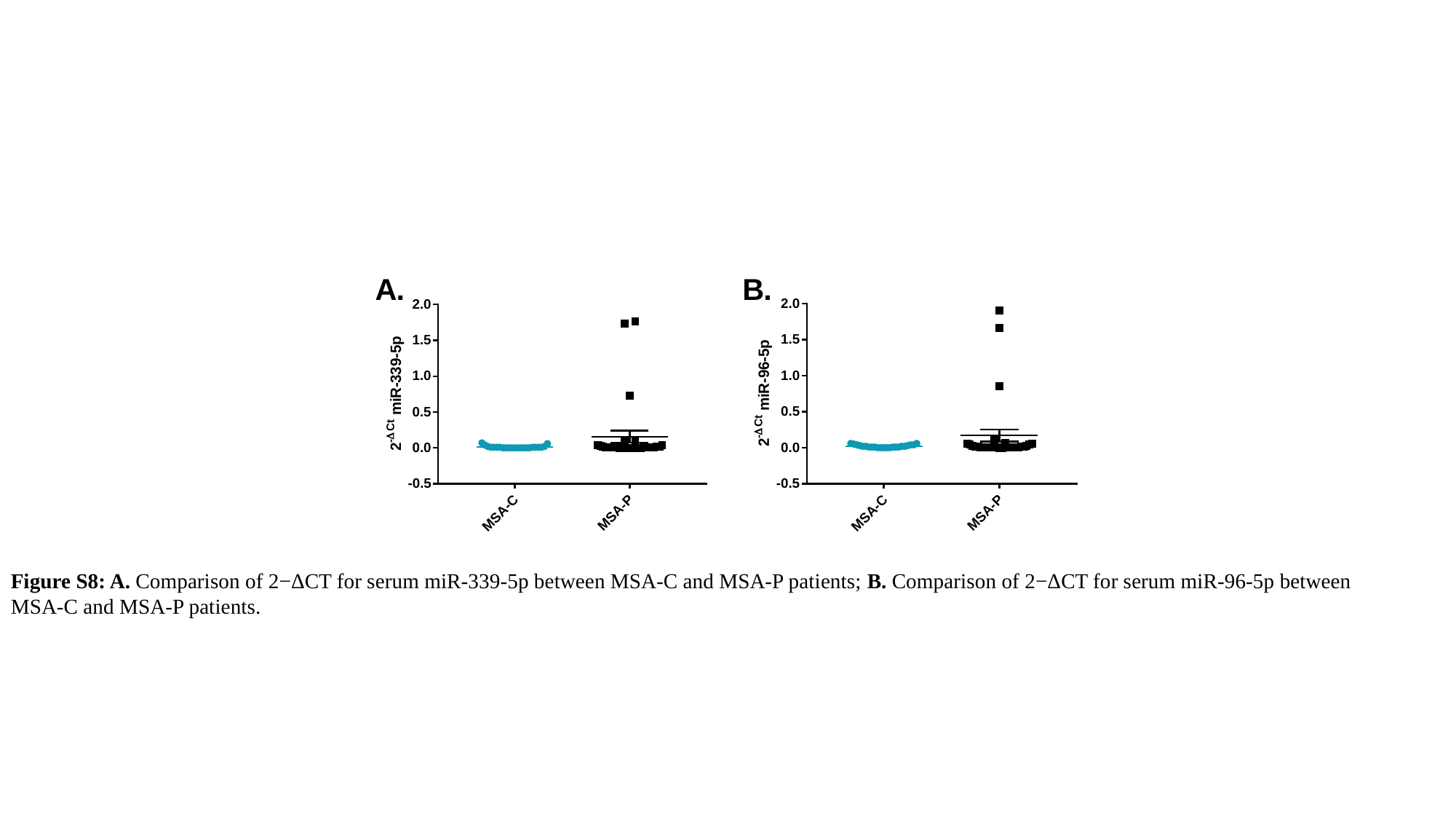

Figure S8: A. Comparison of 2−ΔCT for serum miR-339-5p between MSA-C and MSA-P patients; B. Comparison of 2−ΔCT for serum miR-96-5p between MSA-C and MSA-P patients.

## Slide 10
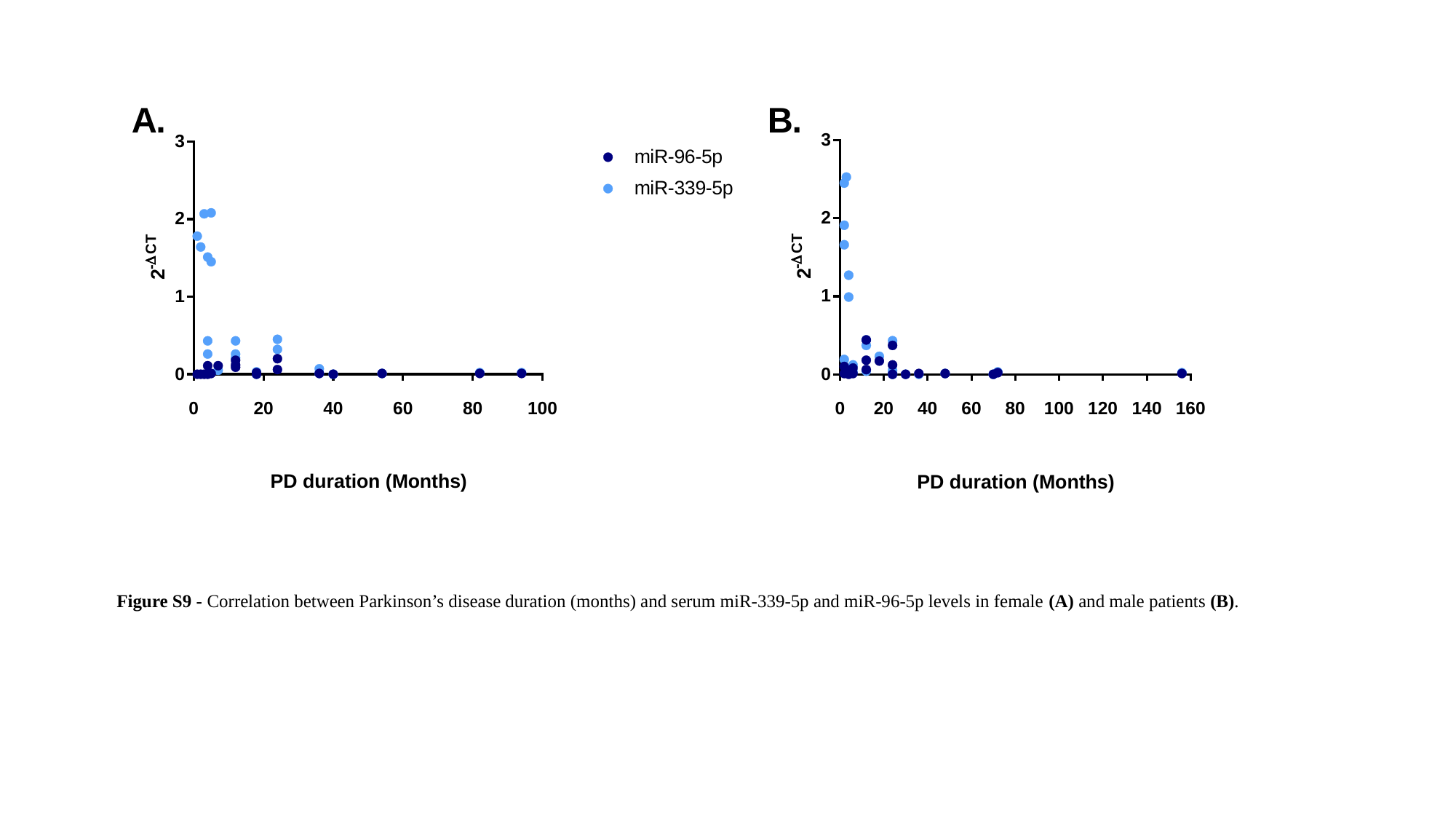

Figure S9 - Correlation between Parkinson’s disease duration (months) and serum miR-339-5p and miR-96-5p levels in female (A) and male patients (B).

## Slide 11
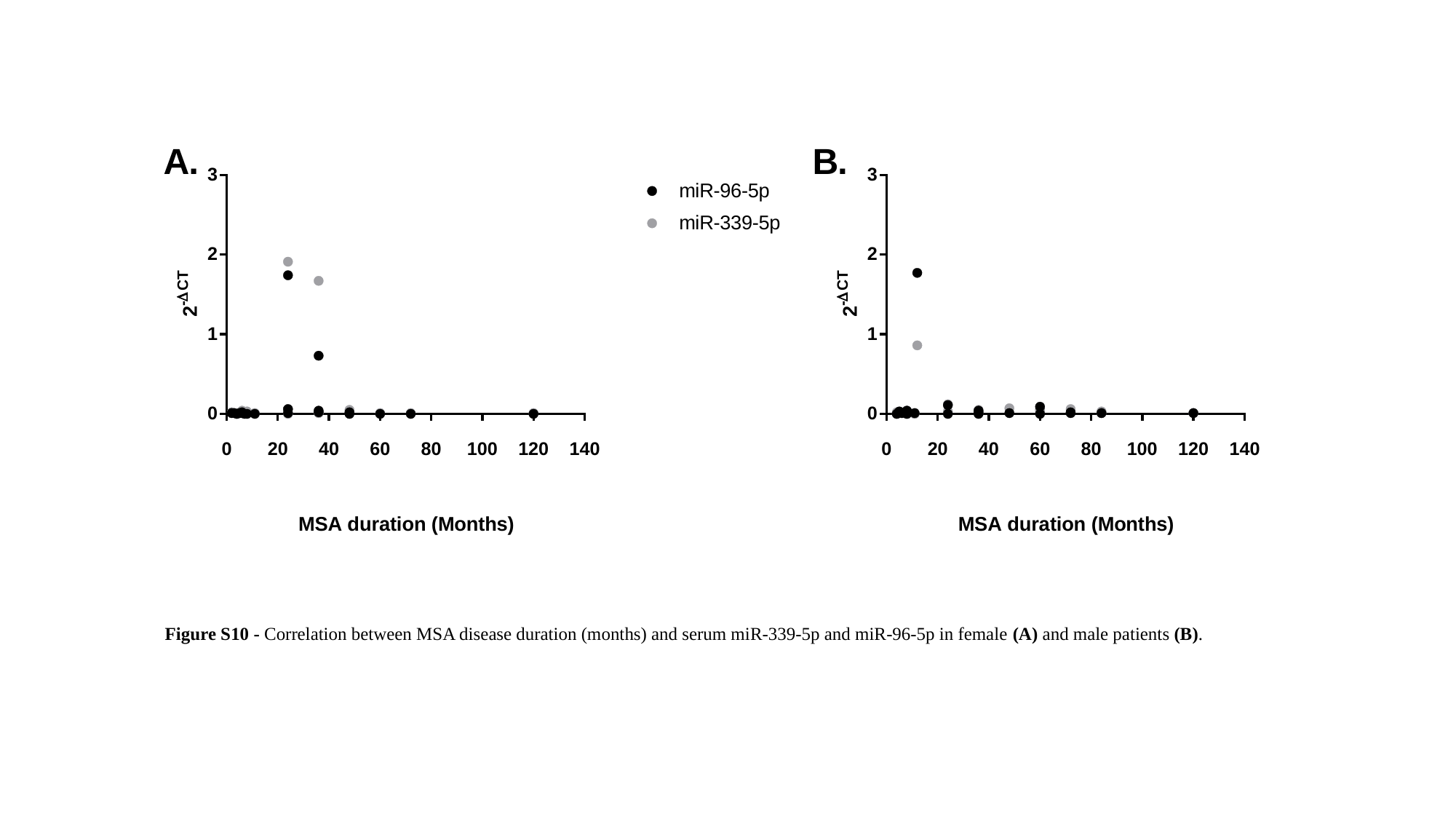

Figure S10 - Correlation between MSA disease duration (months) and serum miR-339-5p and miR-96-5p in female (A) and male patients (B).

## Slide 12
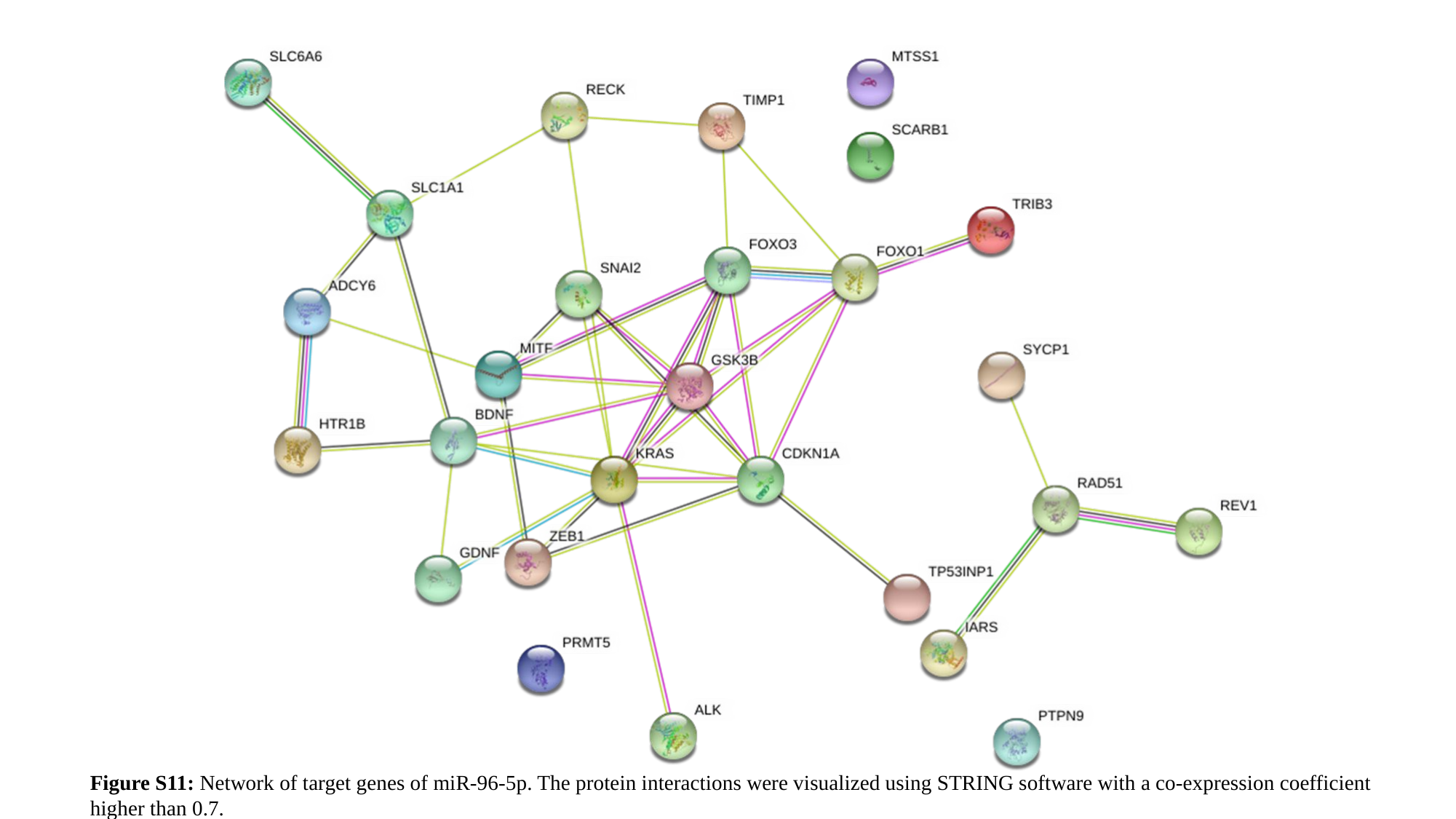

Figure S11: Network of target genes of miR-96-5p. The protein interactions were visualized using STRING software with a co-expression coefficient
higher than 0.7.

## Slide 13
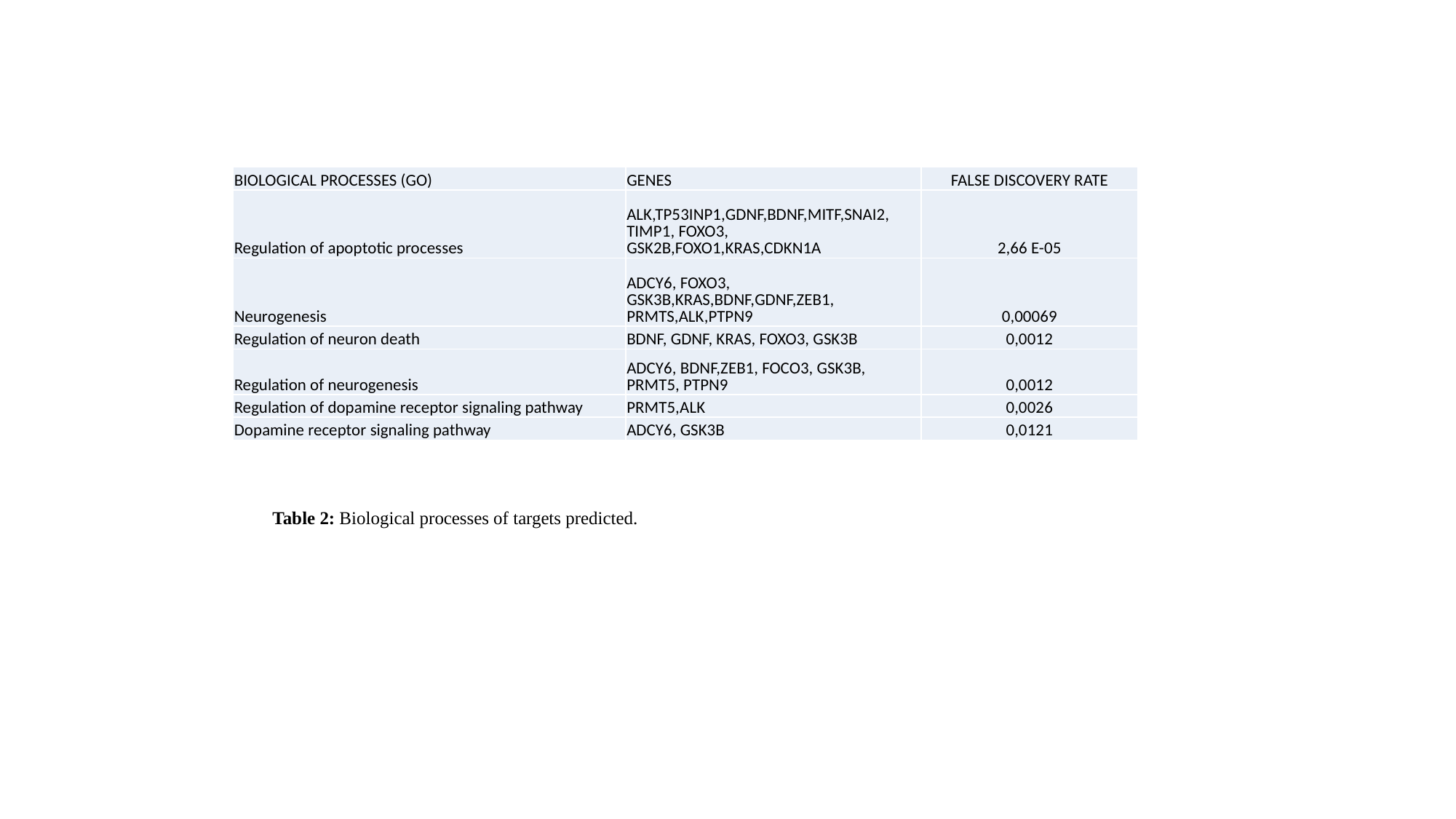

| BIOLOGICAL PROCESSES (GO) | GENES | FALSE DISCOVERY RATE |
| --- | --- | --- |
| Regulation of apoptotic processes | ALK,TP53INP1,GDNF,BDNF,MITF,SNAI2, TIMP1, FOXO3, GSK2B,FOXO1,KRAS,CDKN1A | 2,66 E-05 |
| Neurogenesis | ADCY6, FOXO3, GSK3B,KRAS,BDNF,GDNF,ZEB1, PRMTS,ALK,PTPN9 | 0,00069 |
| Regulation of neuron death | BDNF, GDNF, KRAS, FOXO3, GSK3B | 0,0012 |
| Regulation of neurogenesis | ADCY6, BDNF,ZEB1, FOCO3, GSK3B, PRMT5, PTPN9 | 0,0012 |
| Regulation of dopamine receptor signaling pathway | PRMT5,ALK | 0,0026 |
| Dopamine receptor signaling pathway | ADCY6, GSK3B | 0,0121 |
Table 2: Biological processes of targets predicted.

## Slide 14
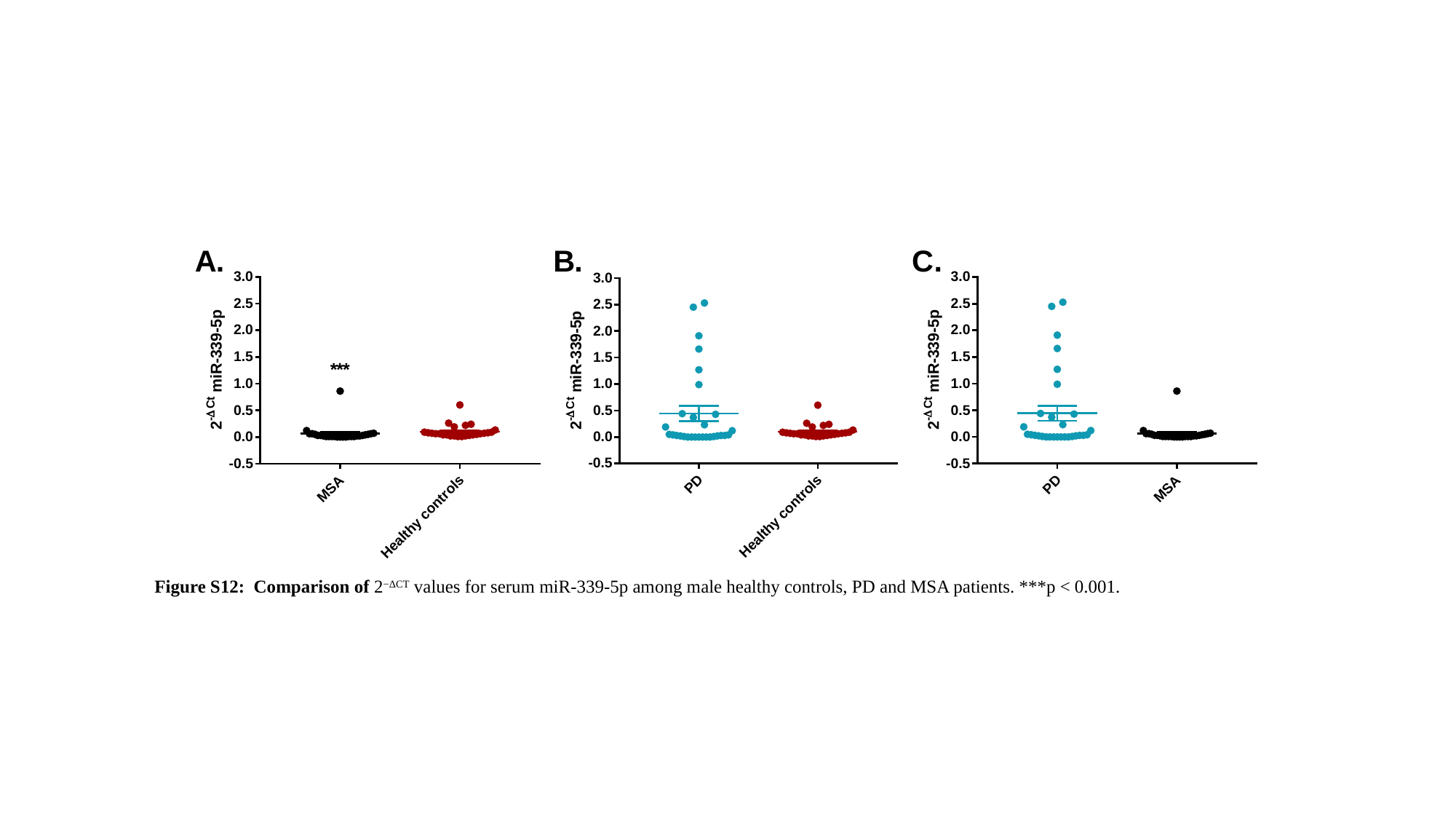

Figure S12: Comparison of 2−ΔCT values for serum miR-339-5p among male healthy controls, PD and MSA patients. ***p < 0.001.

## Slide 15
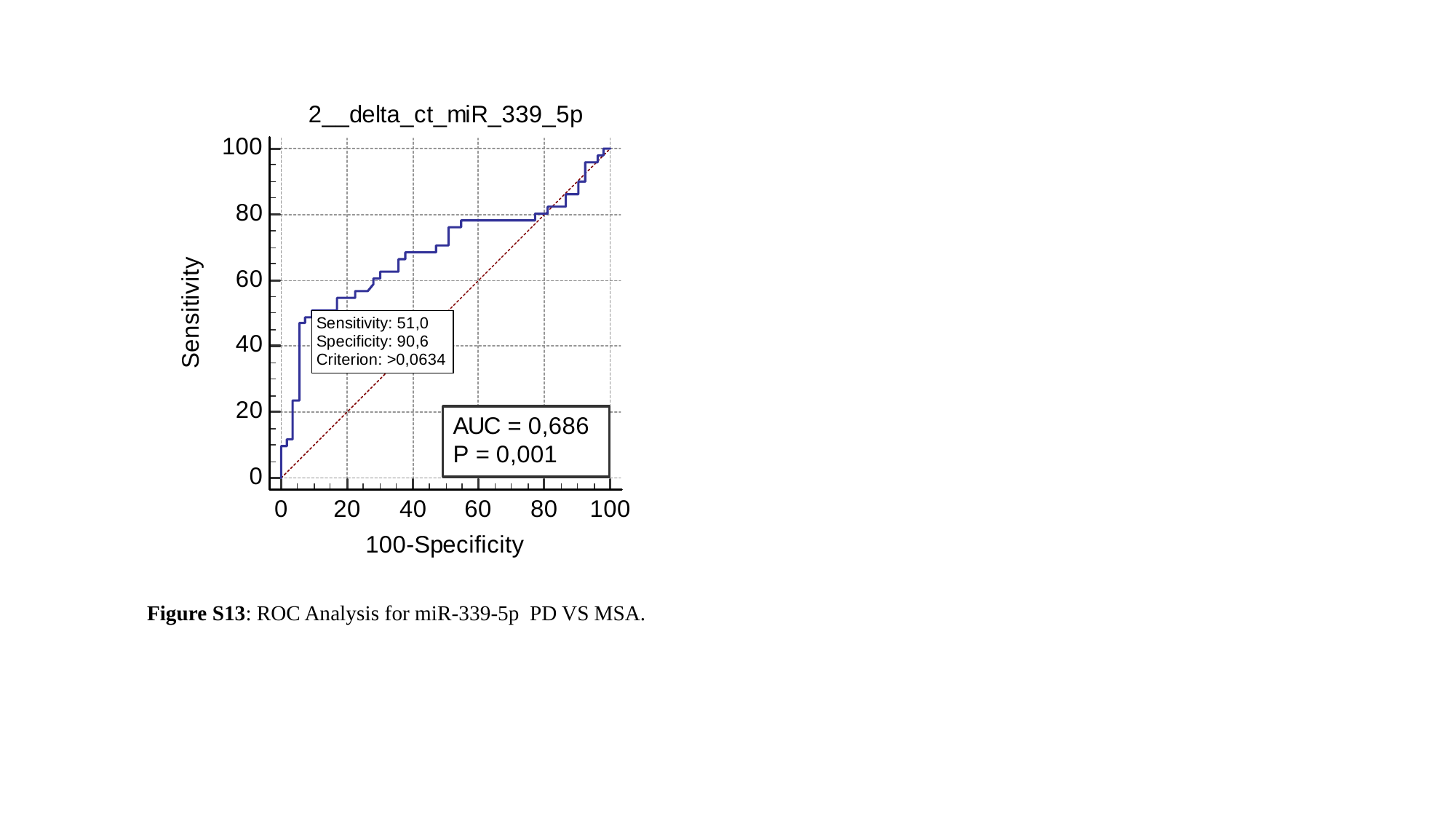

Figure S13: ROC Analysis for miR-339-5p PD VS MSA.

## Slide 16
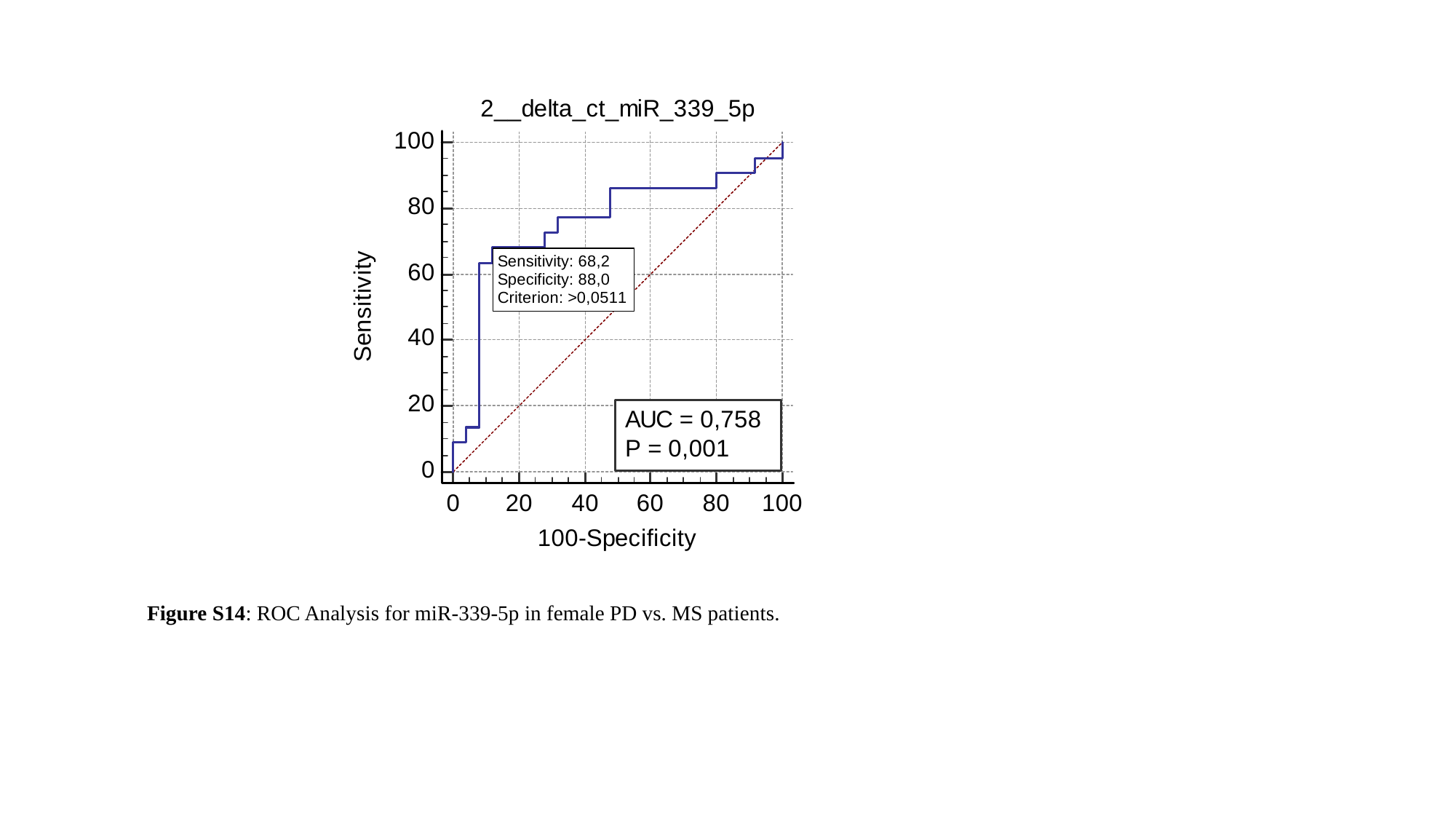

Figure S14: ROC Analysis for miR-339-5p in female PD vs. MS patients.
